# Anti-Inflammatory Effects of a *Stauntonia hexaphylla* Fruit Extract in Lipopolysaccharide-Activated RAW-264.7 Macrophages and Rats by Carrageenan-Induced Hind Paw Swelling

**DOI:** 10.3390/nu10010110

**Published:** 2018-01-22

**Authors:** Jaeyong Kim, Heesook Kim, Hakjoon Choi, Ara Jo, Huwon Kang, Hyojeong Yun, Sojeong Im, Chulyung Choi

**Affiliations:** Jeonnam Institute of Natural Resources Research, Jangheung-gun, Jeollanamdo 59338, Korea; jykim761217@gmail.com (J.K.); awoainia@naver.com (H.K.); ohchj12@naver.com (H.C.); joara9153@naver.com (A.J.); whk0081@naver.com (H.K.); hyojong21@hanmail.net (H.Y.); sojungbalm@naver.com (S.I.)

**Keywords:** *Stauntonia hexaphylla*, anti-inflammatory, NF-κB, neochlorogenic acid, cryptochlorogenic acid, chlorogenic acid, carrageenan-induced paw edema

## Abstract

The fruit of *Stauntonia hexaphylla* is commonly used as a traditional anthelmintic in Korea, Japan, and China. However, its anti-inflammatory activity and the underlying mechanisms have not been studied systematically. In the present study, we examined the anti-inflammatory activities of an aqueous extract of *S. hexaphylla* fruit (SHF) in lipopolysaccharide (LPS)-activated RAW 264.7 cells. The SHF extract contained anti-inflammatory compounds, such as neochlorogenic acid, chlorogenic acid, and cryptochlorogenic acid. The extract inhibited protein levels of inducible nitric oxide synthase and the activity of cyclooxygenase enzyme, with concomitant reductions in the production of nitric oxide and prostaglandin E_2_ in LPS-activated RAW 264.7 cells. Additionally, the SHF extract reduced the production of pro-inflammatory cytokines, including tumor necrosis factor-α, interleukin (IL)-1β, and IL-6. The SHF extract attenuated LPS-induced nuclear factor-κB (NF-κB) activation by decreasing the phosphorylation of its inhibitor, IκBα. Furthermore, the SHF extract showed a significant anti-inflammatory effect in vivo by reducing the volume of carrageenan-induced paw edema in rats. Our results suggest that the SHF extract exerts potential anti-inflammatory properties against LPS-activated RAW 254.7 cells, and in an animal model of inflammation.

## 1. Introduction

For a long time, plants have been a major source of pharmacologically-active substances [1,2]. Plant extracts possess various biological compounds and have been used commonly for the treatment of inflammatory diseases [3,4]. In a recent study, the anti-inflammatory activity of several plant extracts was demonstrated scientifically [5,6,7,8,9]. Therefore, various plant-derived remedies have recently received great interest because of their diverse bioactive constituents and relatively low toxicity in the treatment of various medical conditions, including inflammation-related ailments [3,10].

*Stauntonia hexaphylla* is an evergreen climber with palmate leaves, small bell-shaped flowers, and edible fruits. The plant is widely distributed in Korea, Japan and China [11,12]. The known chemical compounds of *S. hexaphylla* include triterpenoids, glucosides, flavonoids, and phenylpropanoids [13]. However, the anti-inflammatory effects and the mechanism of the *S. hexaphylla* fruit (SHF) extracts have not been reported.

Inflammation is characterized by well-coordinated events in response to harmful stimuli, such as injury, infection, and irritants [14,15]. However, uncontrolled and chronic inflammation can lead to several diseases, such as autoimmune diseases, rheumatoid arthritis, asthma, and inflammatory bowel disease [16,17,18,19]. Non-steroidal anti-inflammatory drugs (NSAIDs) are potent synthetic drugs, widely used for the treatment of inflammatory diseases. Unfortunately, currently available NSAIDs have not been completely successful in clinical applications because of their serious side effects, such as gastric lesions, renal damage, bronchospasm, and cardiac abnormalities [20]. Therefore, there is a global search for new anti-inflammatory drugs as alternatives to NSAIDs.

Macrophages play an important role in the inflammatory processes and produce inflammatory mediators, such as nitric oxide (NO) and prostaglandin E_2_ (PGE_2_), which are generated by activated inducible NO synthase (iNOS) and cyclooxygenase-2 (COX-2), respectively [21]. In addition, macrophages produce various cytokines, such as interleukin-1β (IL-1β), IL-6, and tumor necrosis factor-α (TNF-α), when activated by appropriate stimuli [22]. Activated macrophages thus play pivotal roles in inflammatory diseases via excess production of inflammatory mediators, such as NO and PGE_2_, as well as pro-inflammatory cytokines, to promote the inflammatory response [23].

The expression of inflammatory mediators is induced by nuclear factor-κB (NF-κB), a transcription factor that mediates major inflammatory molecular mechanisms [23]. According to previous reports, plant extracts exert anti-inflammatory effects by inhibiting the NF-κB pathways [24,25]. In macrophages, NF-κB is a transcriptional regulator of inflammatory responses involving the expression of pro-inflammatory cytokines and mediators [26,27]. In cells, inactive NF-κB is localized in the cytoplasm by binding to inhibitor of κB (IκB). When cells are stimulated with lipopolysaccharide (LPS), IκB is degraded, releasing NF-κB, which is then translocated from the cytosol to the nucleus [28].

In the present study, we aimed to ascertain the anti-inflammatory properties of an SHF extract against LPS-activated RAW 254.7 cells, and in an animal model of inflammation.

## 2. Materials and Methods

### 2.1. Plant Materials and Extraction

**Figure 1:**
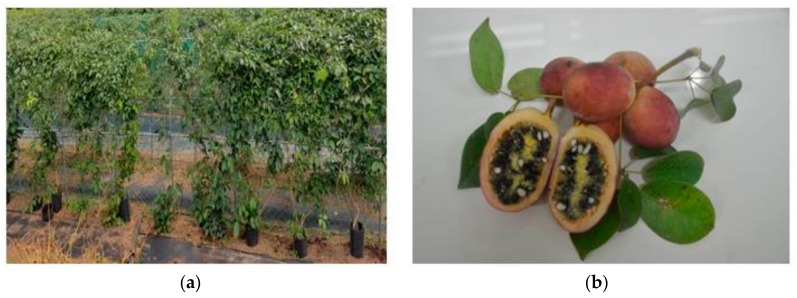
Photographs of *Stauntonia hexaphylla* fruit; (**a**) Cultivated fields (**b**) Fruits.

SHF were collected from cultivated fields in Jangheung-gu, Jeonnam, Korea (Figure 1). The plant was identified by a professor of botany from Chosun University, Gwangju, Korea. SHF (10 kg) were extracted in 10 volumes (*w*/*v*) of distilled water for 3 h at 100 °C. The process was repeated three times and the filtrates were pooled. The resulting extract was concentrated under reduced pressure (Buchi, Flawil, Switzerland) at 40 °C and lyophilized at −50 °C.

### 2.2. High-Performance Liquid Chromatography (HPLC) Analysis

HPLC analysis of the SHF extract was performed using a Waters HPLC system (Milford, MA, USA). The system comprised a binary pump (1525), a photodiode array detector (2998), and an auto injector (2707). A Sunfire C_18_ (250 mm × 4.6 mm, 5 µm, Waters) column was used and the detection wavelengths were set at 254 and 320 nm. The column thermostat was maintained at 35 °C. Mobile phase A was methanol (JT baker, Deventer, Holland) and mobile phase B was water containing 0.1% formic acid (Sigma-Aldrich, St. Louis, MO, USA). SHF extract was standardized, based on neochlorogenic acid, chlorogenic acid, and cryptochlorogenic acid (Sigma-Aldrich, St. Louis, MO, USA). The elution profile was: 0–15 min, 25% A; 15–25 min, 25–40% A; 25–35 min, 40% A; 35–45 min, 40–50% A; 45–55 min, 50–60% A; 55–68 min, 60–100% A; 68–76 min, 100% A; 76–81 min, 100–25% A; and 81–85 min, 25% A. The flow rate was 1 mL/min and the injection volume was 10 µL.

### 2.3. Cell Culture

RAW 264.7 macrophages were obtained from the American Type Culture Collection (ATCC, Manassas, VA, USA) and were cultured in Dulbecco’s modified Eagle’s medium (DMEM; GIBCO, Grand Island, NY, USA); supplemented with 10% heat-inactivated fetal bovine serum (GIBCO, NY, USA), penicillin (100 units/mL), streptomycin (100 µg/mL), l-glutamine (4.5 mg/mL), and glucose (4.5 mg/mL). Cells were incubated at 37 °C in a humidified atmosphere containing 5% CO_2_ and 95% air.

### 2.4. Cell Viability

Cell viability was determined colorimetrically using the 3-(4,5-dimethylthiazol-2yl)-2,5-diphenyl-2*H*-tetrazolium bromide (MTT; Sigma-Aldrich, St. Louis, MO, USA) assay. RAW 264.7 cells were seeded at 1 × 10^4^ cells/well in 96-well plates. After 2 h, the SHF extract (50, 100 and 200 µg/mL) was added to the cells and later treated with LPS (1 µg/mL) at 37 °C for 18 h. Twenty μL of MTT solution (5 mg/mL) was added to each well, and the plates were incubated for 4 h at 37 °C. The supernatants were then aspirated and the formazan crystals in each well were dissolved in 200 μL of dimethyl sulfoxide (DMSO) for 30 min at 37 °C. The optical density at 570 nm was read on a microplate reader (Bio-Rad, Hercules, CA, USA).

### 2.5. Determination of NO and PGE_2_ Production

RAW 264.7 macrophages (5 × 10^5^ cells/mL), plated onto 48-well plates, were pre-incubated for 2 h. Then, the SHF extract (50, 100 and 200 µg/mL) was added to the cells and later treated with LPS (1 µg/mL) at 37 °C for 18 h. The level of NO production was determined by assaying the culture supernatants for nitrite, which is the stable product of a reaction between NO and molecular oxygen, using the Griess reagent, as described previously [29].

PGE_2_ levels were measured using RAW 264.7 cells (1 × 10^5^ cells/well) that were incubated with SHF extract (50, 100 and 200 µg/mL) and later treated with LPS (1 µg/mL) for 18 h. The PGE_2_ level in the supernatants was estimated using a specific enzyme immunoassay kit, according to the manufacturer’s specifications (Cayman Chemicals, San Diego, CA, USA).

### 2.6. Measurement of COX Enzyme Activity

COX enzyme activity was measured using a fluorescent activity assay kit (Cayman Chemicals, San Diego, CA, USA), according to the manufacturer’s specifications. Briefly, RAW 264.7 cells (1 × 10^5^ cells/well) were incubated with SHF extract (50, 100 and 200 µg/mL) and later LPS (100 ng/mL) for 18 h. Supernatants were then used for the determination of COX enzyme activity.

### 2.7. Measurement of Cytokine Production

Cells (1 × 10^6^ cells/well) in 48-well plates were treated with SHF (50, 100 and 200 µg/mL) and later treated with LPS (1 µg/mL) at 37 °C for 18 h. The supernatants were subsequently employed for the pro-inflammatory cytokine (TNF-α, interleukin (IL)-1β, and IL-6) assays using a mouse enzyme-linked immunosorbent assay (ELISA) kit (R&D Systems, Minneapolis, MN, USA).

### 2.8. Western Blot Analysis

Western blot analysis was performed by lysing the cells in radioimmunoprecipitation assay buffer (25 mM Tris-HCl, pH 7.4, 150 mM NaCl, 1% NP-40, 1% sodium deoxycholate, 0.1% sodium dodecyl sulfate) containing a protease inhibitor mixture. The protein concentration was determined using the Bradford assay; the absorbance of the mixture at 595 nm was determined with an ELISA plate reader. An equal amount of protein for each sample was resolved using 8–10% sodium dodecyl sulfate-polyacrylamide gel electrophoresis. Proteins were then transferred electrophoretically onto a polyvinylidene difluoride membrane (Roche, Mannheim, Germany). After blocking nonspecific binding sites with 5% skim milk in TBST buffer (0.1% Tween 20 in PBS), the membrane was incubated overnight with specific primary antibodies (anti-rat iNOS, anti-rat COX-1, anti-rat COX-2, anti-mouse NF-κB, anti-mouse p-IκBα, anti-mouse β-actin, anti-mouse PCNA) at 4 °C. The membrane was then incubated with the secondary antibody (goat anti-rat IgG, goat anti-mouse IgG). Antibodies were purchased from Cell signaling Biotechnology, Inc. (CA, USA). The immunoactive protein bands were performed with enhanced chemiluminescense (ECL) detection (Animal Genetics, St. Austell, UK). The blots were reported with an anti-β action antibody as a control for protein loading.

### 2.9. Animals

Male Sprague–Dawley rats, 5–6 weeks of age, weighing 180–200 g, were purchased from Samtako (Osan-si, Gyeonggi-do, Korea). The animal room environment was maintained at a temperature of 22 ± 3 °C, relative humidity of 50 ± 20%, ventilation of 10–15 air changes/h, and a 12-h light/dark cycle. Animals were provided with food and water ad libitum. This study was approved by the Institutional Animal Care and Use Committee (IACUC) at Jeollanamdo Institute for Natural Resources Research (approval No. JINR1701). All animal experiments were conducted in accordance with the IACUC guidelines.

### 2.10. Carrageenan-Induced Paw Edema in Rats

Carrageenan-induced rat hind paw edema was induced and evaluated, as described previously [30]. Briefly, the group treated with SHF extract was administered 50, 100 and 200 mg/kg for 3 days before the carrageenan injection (1% carrageenan suspended in saline), whereas celecoxib (60 mg/kg, positive control, p.o.) was administered 1 h before the carrageenan injection. Subsequently, 1 h after treatment, 1% carrageenan was injected into the right hind paw of each rat. The left paw was used as a control (0.1 mL saline injected). Paw edema volume was measured before, and at 1, 3 and 5 h after the carrageenan injection, using a plethysmometer (37140, Ugo Basile, Comerio, Italy).

### 2.11. Statistical Analysis

The results are expressed as means ± SD. Comparisons between groups were performed using a one-way analysis of variance (ANOVA). Differences between individual treatment groups were compared using Dunnett’s test. Statistical significance was set at *p* < 0.05 and *p* < 0.01, and the statistical analyses were performed using GraphPad Prism software, version 5.0 (GraphPad Software, Inc., La Jolla, CA, USA).

## 3. Results

### 3.1. Analysis of the SHF Extract for Compounds with Anti-Inflammatory Activity

**Figure 2:**
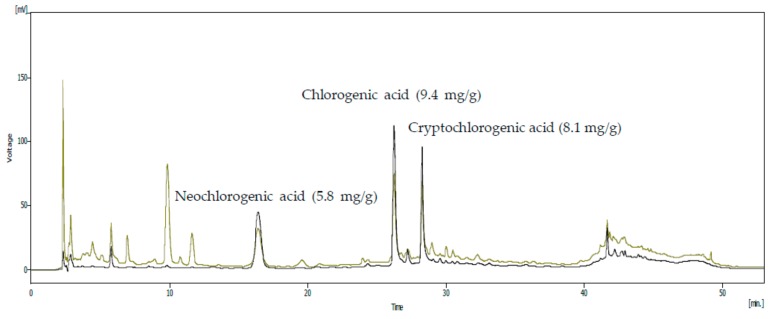
High-performance liquid chromatography of the *Stauntonia hexaphylla* fruit extract. The mobile phase consisted of solvents A (methanol) and B (0.1% formic acid), run at a flow rate of 1.0 mL/min. Elution conditions were as follows: 0–15 min, 25% A; 15–25 min, 25–40% A; 25–35 min, 40% A; 35–45 min, 40–50% A; 45–55 min, 50–60% A; 55–68 min, 60–100% A; 68–76 min, 100% A; 76–81 min, 100–25% A; and 81–85 min, 25% A. The sample injection volume was 10 µL. Optimum HPLC separation was achieved at 35 °C and monitored at 254 and 320 nm. AU: arbitrary units.

To confirm the presence of active anti-inflammatory compounds in the SHF extract, HPLC was employed to identify the chemical components. The following phenolic compounds were identified (Figure 2): neochlorogenic acid, chlorogenic acid, and cryptochlorogenic acid; there were approximately 5.8, 9.4 and 8.1 mg/g of extract, respectively.

### 3.2. Effect of the SHF Extract on the Viability of RAW 264.7 Cells

**Figure 3:**
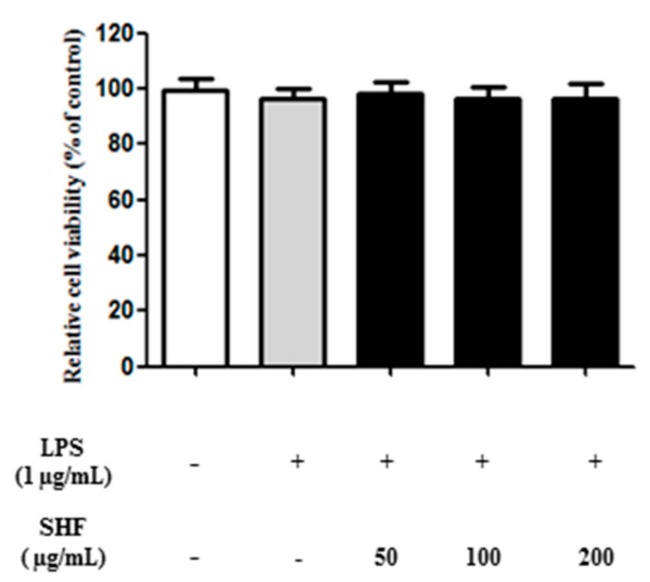
Effect of the *Stauntonia hexaphylla* fruit (SHF) extract on cell viability. RAW 264.7 cells were treated with 50–200 μg/mL of the SHF extract and later treated with lipopolysaccharide (LPS) (1 μg/mL) for 18 h. Cytotoxicity was estimated by the MTT assay.

To obtain a suitable concentration range for investigating the effects of the SHF extract on RAW 264.7 cell viability, cells were treated with concentrations ranging from 50 to 200 µg/mL and later treated with lipopolysaccharide (LPS) (1 μg/mL) for 18 h. There were no significant alterations in cell viability following SHF extract treatment at these concentrations (Figure 3).

### 3.3. Effect of the SHF Extract on LPS-Induced Production of NO and PGE_2_ in RAW 264.7 Cells

**Figure 4:**
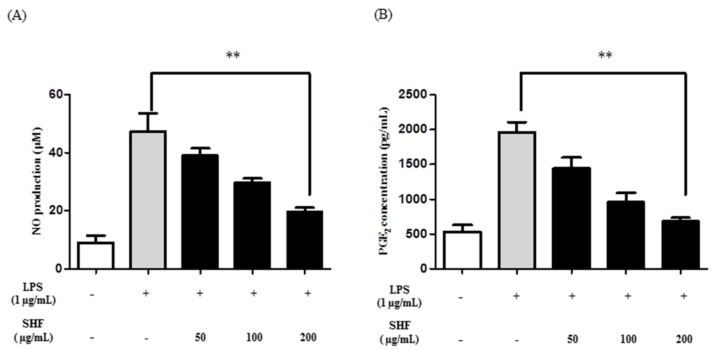
Effect of the *Stauntonia hexaphylla* fruit (SHF) extract on (**A**) the production of nitric oxide (NO) and (**B**) PGE_2_ in lipopolysaccharide (LPS)-stimulated RAW 264.7 cells. Cells were treated with 50, 100 and 200 µg/mL of the SHF extract and later treated with LPS (1 µg/mL) for 18 h. Data are presented as means ± SD. ** *p* < 0.01 compared to the LPS-stimulated group (ANOVA followed Dunnett’s test).

RAW 264.7 cells were treated with 50, 100 and 200 µg/mL of the SHF extract, with and without stimulation with LPS, to determine whether the extract inhibited the production NO and PGE_2_. The LPS increased NO and PGE_2_ production, compared to that in untreated cells. The SHF extract inhibited these increases at a dose of 200 µg/mL (Figure 4).

### 3.4. Effect of the SHF Extract on LPS-Induced COX Enzyme Activity and the Expression of iNOS Protein in RAW 264.7 Cells

**Figure 5:**
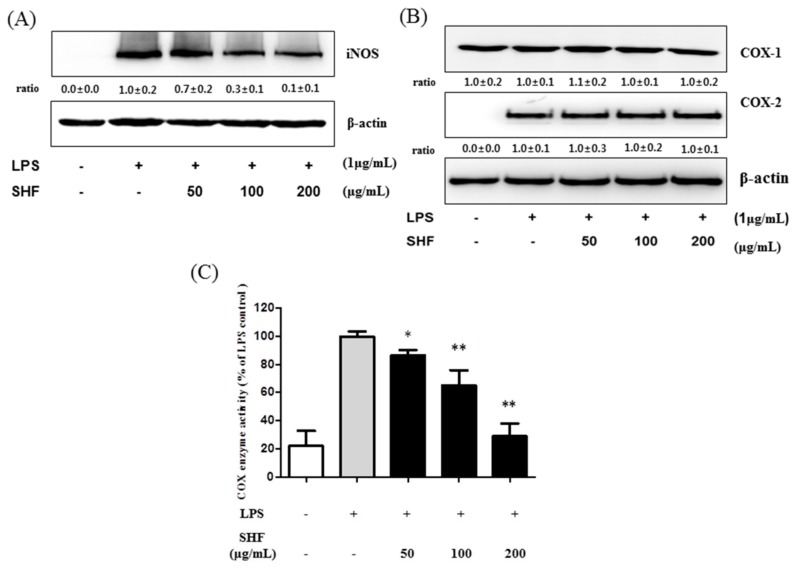
Effect of the *Stauntonia hexaphylla* fruit (SHF) extract on lipopolysaccharide (LPS)-induced inducible nitric oxide synthase (iNOS) protein, cyclooxygenase-2 (COX-2) protein expression and cyclooxygenase (COX) enzyme activity in RAW 264.7 cells. Cells were treated with 50, 100 and 200 µg/mL of the SHF extract and later treated with LPS (1 µg/mL) for 18 h. (**A**) iNOS protein levels were assessed by Western blotting; (**B**) COX-2 protein levels were assessed by Western blotting; (**C**) COX enzyme activity was assessed by a fluorescent assay. Data are presented as means ± SD. * *p* < 0.05 and ** *p* < 0.01 compared to the LPS-stimulated group (ANOVA followed Dunnett’s test).

To analyze whether the inhibitory effect of the SHF extract on NO and PGE_2_ production was related to the modulation of iNOS and COX-2 expression, Western blot analyses were performed. SHF extract, at concentrations of 100 and 200 µg/mL, caused a significant decrease in iNOS protein expression in LPS-stimulated cells (Figure 5A), but SHF extract did not affect the expression of COX-2 protein (Figure 5B). The effect of SHF extract on the activity of COX enzyme was determined by a fluorescent assay kit. As shown in Figure 5C, the SHF extract decreased COX enzyme activity compared to cells treated with LPS alone.

### 3.5. Effect of the SHF Extract on LPS-Induced Production of Pro-Inflammatory Cytokines in RAW 264.7 Cells

**Figure 6:**
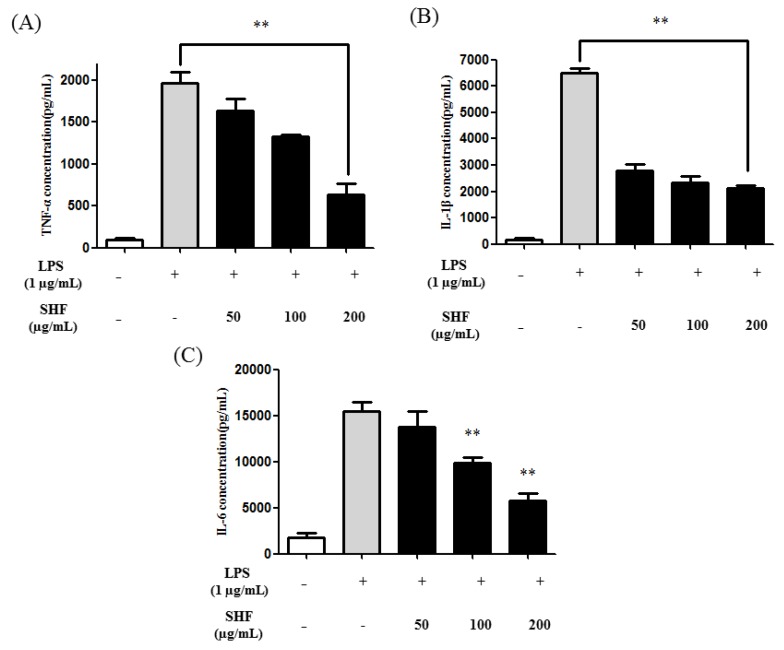
Effect of the *Stauntonia hexaphylla* fruit (SHF) extract on the production of pro-inflammatory cytokines in RAW 264.7 cells. The production of (**A**) tumor necrosis factor (TNF)-α; (**B**) interleukin (IL)-1β; and (**C**) interleukin IL-6 was assayed in the culture medium of cells treated with 50, 100 and 200 µg/mL of the SHF extract and later treated with LPS (1 µg/mL) for 18 h. The concentrations of TNF-α, IL-1β, and IL-6 in the supernatants were determined by enzyme-linked immunosorbent assays. Data are presented as means ±SD. ** *p* < 0.01 compared to the LPS-stimulated group (ANOVA followed Dunnett’s test).

Several cytokines, such as IL-1β, IL-6, and TNF-α, are potent activators of NO production in macrophages [21]. Figure 6 shows that LPS clearly enhanced the production of TNF-α, IL-1β, and IL-6 in RAW 264.7 cells. In contrast, the SHF extract significantly inhibited their production at a dose of 200 µg/mL (Figure 6).

### 3.6. Effect of the SHF Extract on LPS-Induced NF-κB Activity and IκB Phosphorylation in RAW 254.7 Cells

**Figure 7:**
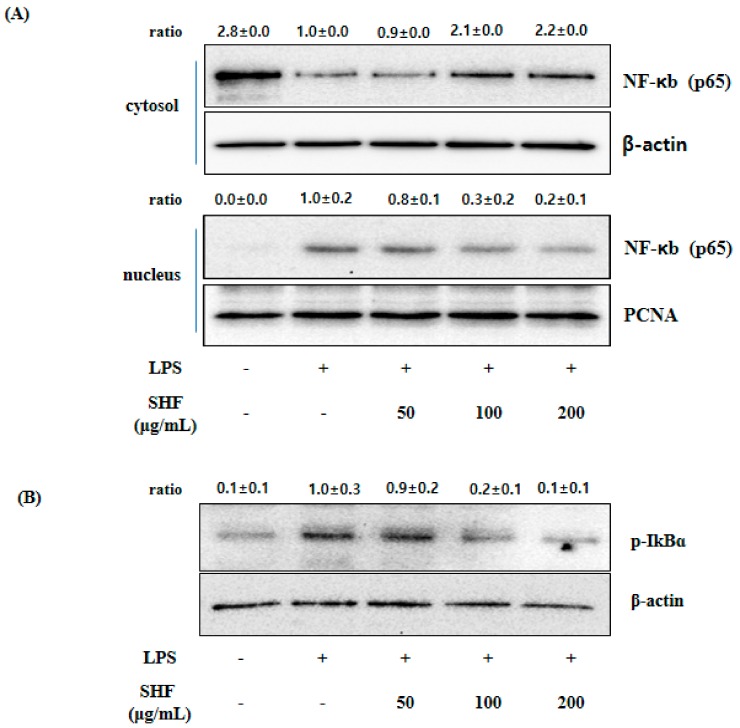
Effect of the *Stauntonia hexaphylla* fruit (SHF) extract on nuclear factor (NF)-κB translocation in RAW 264.7 cells. Cells were treated with 50, 100 and 200 µg/mL of the SHF extract and later treated with LPS (1 µg/mL) for 18 h. After treatment, nuclear and cytosolic extracts were prepared, and equal amounts of proteins were separated by Western blot analyses. (**A**) NF-κB protein expression was determined using an antibody specific for p65; (**B**) Samples were treated as described for Figure 4, and phosphorylation of inhibitor of κB (IκB)α was analyzed by Western blotting.

NF-κB is a well-known and important transcription factor that regulates pro-inflammatory mediators’ synthesis, such as iNOS, IL-6, and TNF-α [26]. Figure 7 shows that the SHF extract concentration dependently inhibited the translocation of NF-κB from the cytosol to the nucleus, and inhibited the phosphorylation of IκBα. This resulted in reduced degradation of IκBα.

### 3.7. Effects of the SHF Extract on Carrageenan-Induced Rat Paw Edema

**Figure 8:**
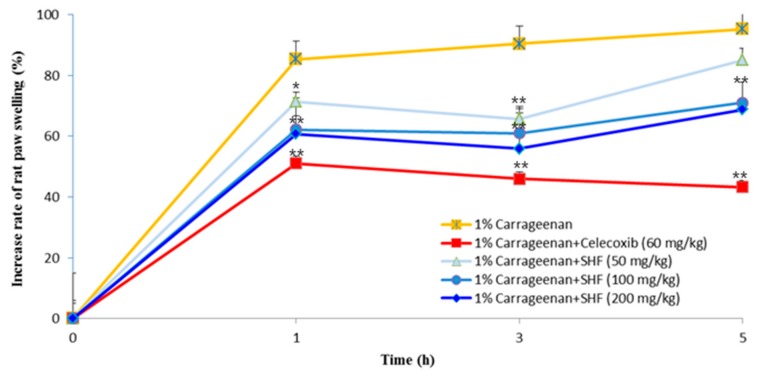
Effect of the *Stauntonia hexaphylla* fruit (SHF) extract on carrageenan-induced rat paw edema. SHF extract was administered in a quantity of 50, 100 and 200 mg/kg for 3 days before the carrageenan injection (1% carrageenan suspended in saline), whereas celecoxib (60 mg/kg, positive control, p.o.) was administered 1 h before the carrageenan injection. Subsequently, 1 h after treatment, 1% carrageenan was injected into the right hind paw of each rat. The left paw was used as a control (0.1 mL saline injected). Edema was measured at 0 h, 1 h, 3 h and 5 h after injection of carrageenan using a plethysmometer (Ugo Basile, Comerio, Italy). Data are presented as means ± SD (*n* = 5). * *p* < 0.05 and ** *p* < 0.01 as compared with 1% Carrageenan group (ANOVA followed Dunnett’s test).

To evaluate the anti-inflammatory effect of the SHF extract in vivo, rat hind paw edema was induced with an intraplantar injection of 1% carrageenan. Celecoxib (positive control, 60 mg/kg) treatment showed a significant inhibitory effect on paw swelling over 1~5 h. The SHF extract (50, 100, and 200 mg/kg) significantly inhibited carrageenan-induced hind paw swelling in the early phase (1 h) as well as effectively inhibiting the increase of paw edema during the late phase (3 h) of inflammation (Figure 8).

## 4. Discussion

Recently, several researchers have focused on plant-derived materials as alternatives for existing anti-inflammatory agents that can cause several side effects. According to our current report, an SHF extract showed potential, non-toxic anti-inflammatory effects in LPS-induced RAW 264.7 cells through inhibiting NF-κB signaling, and in an in vivo model of inflammation. It is important to point out that this study is, to our knowledge, the first to report on the anti-inflammatory effect of an SHF extract.

Macrophages play a significant role in inflammatory diseases that are associated with the excessive production of inflammatory mediators, such as NO, PGE_2_, iNOS, and COX-2, and pro-inflammatory cytokines, including TNF-α, IL-1β, and IL-6 [31]. In our study, the SHF extract (200 μg/mL) significantly inhibited the in vitro production of inflammatory mediators, such as NO, through the suppression of iNOS expression, and PGE_2_ production, through the suppression of COX enzyme activity, at concentrations of 50, 100 and 200 μg/mL.

Additionally, PGE_2_ production is regulated by substrate availability, such as activity of the enzyme COX. COX enzymatic activity is an important aspect in PGE_2_ production [32]. Previous studies reported that *r*-tocopherol, a form of vitamin E, inhibited PGE_2_ production and COX enzyme activity, but showed no effect on the expression of COX protein in macrophages [32,33]. Our results showed that SHF inhibited COX enzyme activity without affecting COX-1 or COX-2 proteins. Thus, SHF extract may be acting by direct inhibition of COX enzyme activity.

The SHF extract also strongly blocked the production of pro-inflammatory cytokines, such as TNF-α, IL-6, and IL-1β, in LPS-activated RAW 264.7 cells.

We evaluated the activation of NF-κB signaling pathways in LPS-activated RAW 264.7 cells. NF-κB is a transcription factor that plays an essential role in inflammation through inducing pro-inflammatory factors, such as iNOS, COX-2, TNF-α, IL-6, and IL-1β. In the inactive state, NF-κB is located in the cytosol as a complex with the inhibitor protein, IκBα. Activation of NF-κB, in response to LPS stimulation, leads to the degradation of IκB, followed by the release and nuclear translocation of NF-κB [28]. Our results show that the SHF extract prevented the nuclear translocation of NF-κB, by suppressing IκBα phosphorylation and degradation in LPS-induced RAW 264.7 cells.

These results indicated a potential role of NF-κB in the SHF extract-mediated inhibition of inflammatory mediators, including NO, PGE_2_, and pro-inflammatory cytokines.

Carrageenan-induced paw edema has been used widely as an animal model of inflammation and is divided into two phases [34]. The early stage (1 h) involves the release of pro-inflammatory compounds, such as histamine and serotonin, while the late phase (>1 h) is due to increased COX-2 and the release of PGE_2_ [35,36]. Based on our findings (Figure 8), the SHF extract significantly and dose-dependently reduced carrageenan-induced inflammation in the early phase as well as effectively inhibiting the increase of paw edema during the late phase (3 h) of inflammation.

The anti-inflammatory compounds in plant extracts are detected at wavelengths of 200–350 nm of HPLC [37]. Investigation into the phytochemical content of the SHF extract revealed the presence of neochlorogenic acid, chlorogenic acid, and cryptochlorogenic acid. These compounds have known anti-inflammatory activity [38,39,40]. Therefore, the anti-inflammatory effect of the SHF extract could be due to the presence of these polyphenol compounds.

## 5. Conclusions

Our results show that the SHF extract significantly inhibited the production of pro-inflammatory mediators, including iNOS, NO, COX enzyme activity, and PGE_2_, and certain cytokines in LPS-activated RAW 264.7 cells. These effects were mediated by blocking NF-κB signal transduction pathways. Additionally, in vivo results supported the role of SHF extract as an anti-inflammatory agent. Taken together, the results of this study not only provide a pharmacological basis for the application of SHF extracts in inflammatory diseases, but can also accelerate the development of a new class of anti-inflammatory agents. In further studies, it will be necessary to investigate other inflammatory models and clinical studies.

## References

[1] Darshan S., Doreswamy R. (2004). Patented antiinflammatory plant drug development from traditional medicine. Phytother. Res..

[2] De las Heras B., Slowing K. (1998). Antiinflammatory and antioxidant activity of plants used in traditional medicine in Ecuador. J. Ethnopharmacol..

[3] Talhouk R.S., Karam C. (2007). Anti-inflammatory bioactivities in plant extracts. J. Med. Food.

[4] Krishnaswamy K. (2008). Traditional Indian spices and their health significance. Asia Pac. J. Clin. Nutr..

[5] Asongalem E.A., Foyet H.S., Ngogang J., Folefoc G.N., Dimo T. (2004). Analgesic and antiinflammatory activities of Erigeron floribundus. J. Ethnopharmacol..

[6] Choi C.Y., Kim J.Y., Kim Y.S., Chung Y.C., Hahm K.S., Jeong H.G. (2001). Augmentation of macrophage functions by an aqueous extract isolated from Platycodon grandiflorum. Cancer Lett..

[7] Talwar S., Nandakumar K., Nayak P.G., Bansal P., Mudgal J., Mor V., Rao C.M., Chamallamudi M.R., Lobo R. (2011). Anti-inflammatory activity of Terminalia paniculata bark extract against acute and chronic inflammation in rats. J. Ethnopharmacol..

[8] Woldesellassie M., Eyasu M., Kelbessa U. (2011). In vivo anti-inflammatory activities of leaf extracts of Ocimum lamiifolium in mice model. J. Ethnopharmacol..

[9] Zhang G.Q., Huang X.D., Wang H., Leung A.K.N., Chan C.L., Fong D.W.F., Yu Z.L. (2008). Anti-inflammatory and analgesic effects of the ethanol extract of *Rosa multiflora* Thunb. hips. J. Ethnopharmacol..

[10] Li Y.C., Xian Y.F., Ip S.P., Su Z.R., Su J.Y., He J.J., Xie Q.F., Lai X.P., Lin Z.X. (2011). Anti-inflammatory activity of patchouli alcohol isolated from Pogostemonis herba in animal models. Fitoterapia.

[11] Park Y.J., Park Y.S., Towantakavanit K., Park J.O., Kim Y.M., Jung K.J., Cho J.Y., Lee K.D., Heo B.G. (2009). Chemical components and biological activity of Stauntonia hexaphylla. Korean J. Plant Resour..

[12] Wang H.B., Mayer R., Rocker G., Yang J.J., Matteson D.S. (1998). A phenolic glycoside and triterpenoids from Stauntonia hexaphylla. Phytochemistry.

[13] Hwang S.H., Kwon S.H., Kim S.B., Lim S.S. (2017). Inhibitory activities of Stauntonia hexaphylla leaf constituents on rat lens aldose reductase and formation of advanced glycation end products and antioxidant. Biomed. Res. Int..

[14] Ryu H.W., Lee S.U., Lee S., Song H.H., Son T.H., Kim Y.U., Yuk H.J., Ro H., Lee C.K., Hong S.T. (2017). 3-methoxy-catalposide inhibited inflammatory effects in lipopolysaccharide-stimulated RAW264.7 macrophages. Cytokine.

[15] Kim K.N., Ko Y.J., Kang M.C., Yang H.M., Roh S.W., Oda T., Jeon Y.J., Jung W.K., Heo S.J., Yoon W.J. (2013). Anti-inflammatory effects of trans-1,3-diphenyl-2,3-epoxypropane-1-one mediated by suppression of inflammatory mediators in LPS-stimulated RAW 264.7 macrophages. Food Chem. Toxicol..

[16] Flavell R.A. (2002). The relationship of inflammation and initiation of autoimmune disease: Role of TNF super family members. Curr. Top. Microbiol. Immunol..

[17] Christodoulou C., Choy E.H. (2006). Joint inflammation and cytokine inhibition in rheumatoid arthritis. Exp. Med..

[18] Bousquet J., Jeffery P.K., Busse W.W., Johnson M., Vignola A.M. (2000). Asthma: From bronchoconstriction to airways inflammation and remodeling. Am. J. Respir. Crit. Care Med..

[19] Danese S., Grisham M., Hodge J., Telliez J.B. (2016). JAK inhibition using tofacitinib for inflammatory bowel disease treatment: A hub for multiple inflammatory cytokines. Am. J. Physiol. Gastrointest. Liver Physiol..

[20] Manrique-Moreno M., Heinbockel L., Suwalsky M., Garidel P., Brandenburg K. (2016). Biophysical study of the non-steroidal anti-inflammatory drugs (NSAID) ibuprofen, naproxen and diclofenac with phosphatidylserine bilayer membranes. Biochim. Biophys. Acta.

[21] MacMicking J., Xie Q.W., Nathan C. (1997). Nitric oxide and macrophage function. Annu. Rev. Immunol..

[22] Korhonen R., Lahti A., Kankaanranta H., Moilanen E. (2005). Nitric oxide production and signaling in inflammation. Curr. Drug Targets Inflamm. Allergy.

[23] Ha Y.M., Chung S.W., Kim J.M., Kim D.H., Kim J.Y., Lee E.K., Lee J., Kim Y.J., Yoo M.A., Jeong K.S. (2010). Molecular activation of NF-κB, pro-inflammatory mediators, and signal pathways in γ-irradiated mice. Biotechnol. Lett..

[24] Song S.M., Ham Y.M., Ko Y.J., Ko E.Y., Oh D.J., Kim C.S., Kim D., Kim K.M., Yoon W.J. (2016). Anti-inflammatory activities of the products of supercritical fluid extraction from Litsea japonica fruit in RAW 264.7 cells. J. Funct. Foods.

[25] Xu J., Zhao Y., Aisa H.A. (2017). Anti-inflammatory effect of pomegranate flower in lipopolysaccharide (LPS)-stimulated RAW264. 7 macrophages. Pharm. Biol..

[26] Makarov S.S. (2000). NF-kappaB as a therapeutic target in chronic inflammation: Recent advance. Mol. Med. Today.

[27] Hanada T., Yoshimura A. (2002). Regulation of cytokine signaling and inflammation. Cytokine Growth Factor Rev..

[28] Liu F., Morris S., Epps J., Carroll R. (2002). Demonstration of an activation regulated NF-kappaB/I-kappaBalpha complex in human platelets. Thromb. Res..

[29] Chu H., Tang Q., Huang H., Hao W., Wei X. (2016). Grape-seed proanthocyanidins inhibit the lipopolysaccharide-induced inflammatory mediator expression in RAW264.7 macrophages by suppressing MAPK and NF-κb signal pathways. Environ. Toxicol. Pharmacol..

[30] Kim J.Y., Yang S.Y., Choi C.Y. (2016). The Evaluation of the effect of herbal extract on osteoarthritis: In vitro and in vivo study. Prev. Nutr. Food Sci..

[31] Bosca L., Zeini M., Traves P.G., Hortelano S. (2005). Nitric oxide and cell viability in inflammatory cells: A role for NO in macrophage function and fate. Toxicology.

[32] Beharka A.A., Wu D., Serafini M., Meydani N. (2002). Mechanism of vitamin E inhibition of cyclooxygenase activity in macrophages from old mice: Role of peroxynitrite. Free Racdic. Biol. Med..

[33] Jiang Q., Elson-Schwab I., Courtemanche C., Ames B.N. (2000). γ-tocopherol and its major metabolite, in contrast to α-tocopherol, inhibited cyclooxygenase activity in macrophages and epithelial cells. Proc. Natl. Acad. Sci. USA.

[34] Masresha B., Makonnen E., Debella A. (2012). In vivo anti-inflammatory activities of Ocimum suave in mice. J. Ethnopharmacol..

[35] Niu X., Li Y., Li W., Hu U., Yao H., Li H., Mu Q. (2014). The anti-inflammatory effects of Caragana tangutica ethyl acetate extract. J. Ethnopharmacol..

[36] Lee S.A., Moon S.M., Choi Y.H., Han S.H., Park B.R., Choi M.S., Kim J.S., Kim Y.H., Kim D.K., Kim C.S. (2017). Aqueous extract of Codium fragile suppressed inflammatory responses in lipopolysaccharide-stimulated RAW264.7 cells and carrageenan-induced rats. Biomed. Pharmacother..

[37] Dong Z.B., Zhang Y.H., Zhao B.J., Li C., Tian G., Niu B., Qi H., Feng L., Shao J.G. (2015). Screening for anti-inflammatory components from Corydalis bungeana Turcz. based on macrophage binding combined with HPLC. BMC Complement. Altern. Med..

[38] Zhen J., Villani T.S., Guo Y., Qi Y., Chin K., Pan M.H., Ho C.T., Simon J.E., Wu Q. (2016). Phytochemistry, antioxidant capacity, total phenolic content and anti-inflammatory activity of Hibiscus sabdariffa leaves. Food Chem..

[39] Seo C.S., Lim H.S., Jeong S.J., Ha H., Shin H.K. (2013). HPLC-PDA analysis and anti-inflammatory effects of Mori Cortex Radicis. Nat. Prod. Commun..

[40] Francisco V., Costa G., Figueirinha A., Marques C., Paulo P., Neves B.M., Lopes M.C., García-Rodríguez C., Cruz M.T., Batista M.T. (2013). Anti-inflammatory activity of Cymbopogon citratus leaves infusion via proteasome and nuclear factor-κB pathway inhibition: Contribution of chlorogenic acid. J. Ethnopharmacol..

